# Digit ratio and autism spectrum disorders in the Avon Longitudinal Study of Parents and Children: a birth cohort study

**DOI:** 10.1136/bmjopen-2014-007433

**Published:** 2015-08-25

**Authors:** Anna Louise Guyatt, Jon Heron, Bernice Le Cornu Knight, Jean Golding, Dheeraj Rai

**Affiliations:** 1 Centre for Academic Mental Health, School of Social and Community Medicine, University of Bristol, Bristol, UK; 2 MRC Integrative Epidemiology Unit, School of Social & Community Medicine, University of Bristol, Bristol, UK; 3 Centre for Child and Adolescent Health, School of Social & Community Medicine, University of Bristol, Bristol, UK; 4 Avon and Wiltshire Partnership NHS Mental Health Trust, Bristol, UK

**Keywords:** EPIDEMIOLOGY, Developmental neurology & neurodisability < PAEDIATRICS, Child & adolescent psychiatry < PSYCHIATRY

## Abstract

**Objectives:**

To investigate whether second-to-fourth digit ratio (2D:4D), a measure commonly used as a proxy for fetal testosterone exposure, is associated with autism spectrum disorders (ASDs), as predicted by the extreme male brain theory of autism.

**Design:**

A birth cohort study.

**Setting:**

The Avon Longitudinal Study of Parents and Children (ALSPAC).

**Participants:**

6015 ALSPAC children with data on digit ratio, at least 1 outcome measure and information on potential confounding variables (parental occupational class, maternal education and age at digit ratio measurement). Digit ratio was measured by the photocopy and calliper method.

**Outcomes:**

ASD diagnosis (cases were identified previously by record linkage or maternal report) and 4 measures that combine optimally within ALSPAC to predict ASD: the Children's Communication Checklist (coherence subscale), the Social and Communication Disorders Checklist, a repetitive behaviour measure, and the Emotionality, Activity and Sociability scale (sociability subscale). These measures were dichotomised, with approximately 10% defined as the ‘risk’ group.

**Results:**

Using logistic regression, we examined the association of 2D:4D with ASDs and 4 dichotomised ASD traits. Covariates were occupational class, maternal education and age at 2D:4D measurement. 2D:4D was not associated with ASDs in males (adjusted OR per 1 SD increase in mean 2D:4D, 0.88 (95% CI 0.65 to 1.21), p=0.435) or females (adjusted OR=1.36 (95% CI 0.81 to 2.28), p=0.245). Similar results were observed after adjustment for IQ. There was 1 weak association between reduced coherence and increased left 2D:4D in males, in the opposite direction to that predicted by the extreme male brain theory (adjusted OR=1.15 (95% CI 1.02 to 1.29), p=0.023). Given multiple comparisons, this is consistent with chance.

**Conclusions:**

In this population-based study, there was no strong evidence of an association between 2D:4D and ASD diagnosis or traits, although the CIs were wide. These results are not consistent with the extreme male brain theory.

**Strengths and limitations of this study**

- To date, this is one of the largest population-based studies examining the relationship between second-to-fourth digit ratio (2D:4D), which is used as a proxy for fetal testosterone exposure, and autism spectrum disorders (ASDs), separately by sex.
- This paper also examines the relationship between 2D:4D and component measures of autism, which may give insight into the less-explored relationship between digit ratio and the individual domains (social and communication, repetitive behaviours) represented in autism.
- In comparison with other small studies based in clinical settings, this study may be less prone to selection bias; the rich phenotype information within the Avon Longitudinal Study of Parents and Children (ALSPAC) allowed us to control for possible confounding variables.
- Despite its relatively large overall sample size compared with many studies, the number of autism cases in this study is still small.
- Cohort studies are prone to bias from attrition, and while the major sociodemographic predictors of attrition were controlled for in this study, it is also possible that children severely affected by ASD may have been less likely to attend clinics where 2D:4D was measured, which could bias our results.

## Introduction

Autism spectrum disorders (ASDs) are pervasive developmental disorders characterised by impaired social communication and reciprocal social interaction; and restricted, repetitive patterns of behaviour, interests or activities.^1^ While twin studies suggest that ASDs are highly heritable, their aetiology remains largely unexplained.^2^ Elucidating the causes of ASD may facilitate earlier diagnosis and enhance the potential for primary prevention. Earlier diagnosis may lead to an improvement in educational and behavioural outcomes, a reduction in comorbid psychiatric symptoms, and a decrease in the emotional and financial stresses created by ASDs for individuals and their families.^3^

Various population cohorts have estimated the prevalence of ASD at approximately 1%.^4–7^ These studies demonstrate a marked gender imbalance, with male-to-female ratio estimates ranging from 2:1^5^ to 9:1,^8^ depending on age of assessment, length of follow-up, and whether studies screen for ASDs or examine data on pre-existing diagnosis. Although diagnostic bias has been proposed as a partial explanation for this disparity between sexes,^9^ ^10^ the consistently observed preponderance among males has generated interest into possible biological mechanisms predisposing to ASDs.

The extreme male brain (EMB) theory is the most popular hypothesis put forward to explain the sex difference in ASD. This theory postulates that children with ASD exhibit an exaggerated form of the male cognitive profile,^11^ and proposes that prenatal androgens are plausible biological candidates. Some evidence from animal studies suggests that testosterone may mediate cognitive differences between the sexes via organisational effects on the brain.^12^ ^13^ Recent evidence supporting the EMB theory found sex steroid levels in amniocentesis samples to be correlated with the diagnosis of ASDs.^14^

The index to ring finger ratio (second-to-fourth digit ratio, 2D:4D) has been widely used as a proxy for fetal testosterone exposure in autism research.^15–25^ An observation supporting a causal association between 2D:4D and fetal testosterone is that 2D:4D has been shown to be sexually dimorphic,^26^ with males generally having lower 2D:4D (ie, a relatively shorter index finger (2D) compared with their ring finger (4D)),^27^ ^28^ although this is not a unanimously reported finding.^25^ This sexual dimorphism is apparent from the first trimester of pregnancy, and appears to be largely static after birth, with most,^29–33^ but not all^25^ studies finding that it is unaffected by pubertal androgen. 2D:4D has also been shown to be sexually dimorphic in endocrine models of elevated (congenital adrenal hyperplasia) and reduced fetal testosterone exposure (complete androgen insensitivity syndrome).^28^ ^34–36^

Although recent reviews have concluded that lower 2D:4D is associated with ASD,^37^ ^38^ many published studies are within relatively small, clinical populations which may be susceptible to selection bias and confounding. We designed a study to examine the association between 2D:4D and ASD and various autistic trait measures in a population-based cohort in the UK. The primary hypothesis being tested was that lower 2D:4D would be associated with ASDs and ASD traits.

## Methods

### Study design and population

The Avon Longitudinal Study of Parents and Children (ALSPAC) is a birth cohort based in Avon, UK. In total, 14 541 pregnant women with expected delivery dates between 1 April 1991 and 31 December 1992 were initially enrolled, and 13 988 children were alive at 1 year.^39^ The study website contains details of all the data that are available through a fully searchable data dictionary: http://www.bris.ac.uk/alspac/researchers/data-access/data-dictionary.^40^ The primary source of data collection was via self-completion questionnaires administered at four points during the prenatal period, then at regular intervals following birth, to both parents and the ‘study child’. Since the age of 7 years, the whole cohort has been invited to a regular ‘focus’ clinic for a variety of hands-on assessments.

### Outcome variables

#### Autism spectrum disorders

Within ALSPAC, the identification of children with ASD has been described elsewhere.^41^ Briefly, a previous record linkage study identified 86 ASD cases in ALSPAC, of which there was evidence for a strict multidisciplinary clinical assessment in 71 individuals, and in 15 others with a diagnosis of ASD recorded in school records.^41^ In a validation study, a consultant paediatrician reviewed the records of the individuals with ASD to confirm concordance with the International Classification of Diseases (ICD) 10 criteria.^41^ In addition to cases identified by record linkage, we further identified individuals with ASD based on maternal report. At 9.5 years, mothers were asked, “Have you ever been told that your child has: autism, Asperger's syndrome or autistic spectrum disorder?” Investigators utilising maternal reports based on similar questions in other cohorts have reported an acceptable validity of identifying ASD using this approach.^42–44^ We cross-validated ASD cases confirmed only by maternal report by studying their association with various autistic trait measures and found strong associations (see online supplementary table S1). Of the 56 ASD cases included in this study, 24 were identified by record linkage, and 32 were identified from maternal reports of ASD diagnosis.

#### Dichotomised ASD traits

A previous factor analysis of 93 measures related to autism also reported four individual measures administered in ALSPAC via parental questionnaires that ‘combined optimally to predict autism’.^45^ The coherence subscale of the Children’s Communication Checklist, measured at 115 months, assesses pragmatic abnormalities in social communication.^46^ The Social and Communication Disorders Checklist, measured at 91 months, measured social skills (described previously).^47^ A Repetitive Behaviour score, measured at 69 months, asked whether the child ‘repeatedly rocks the head or body, has tics or twitches or other unusual behaviour’, and included a question from the Rutter scale on tics, mannerisms or twitches.^48^ The sociability subscale of the Emotionality, Activity and Sociability, measured at 38 months, measures tendency to affiliate and interact with others.^49^ As all the ASD scales considered were highly negatively skewed, and not readily correctable by transformation, each was dichotomised, creating a high-risk (for ASDs) group of as close to 10% of the population as possible.

### Explanatory variable: 2D:4D

Participants were invited to a focus clinic, when 2D:4D was measured. Mean measurement age was 11.75 years. While 2D:4D was measured *after* the outcome measures (between approximately 2 and 8 years later, depending on the outcome), it is widely used as a measurement of the prenatal environment, and therefore we feel justified in using it as our exposure in our analysis.

2D:4D was measured by the photocopy and calliper method: the ventral surface of each palm was placed as flatly as possible onto a photocopier, ensuring that the fingers were apart and the palms pressed firmly onto the glass. The lengths of the second (index) and fourth (ring) fingers were measured using the ‘Mahr digital caliper16 EX’, accurate to 0.01 mm. Each finger measured has a crease at its base: the index finger (2D) has one crease, the ring finger (4D) probably three or four. The most proximal crease was chosen and by eye, the midpoint of this was determined and the distance from this crease to the distal fingertip measured. 2D measurement was divided by 4D measurement, thus giving a 2D:4D measurement for each hand.^40^ To assess the legitimacy of using photocopies to calculate 2D:4D, 57 right and 48 left hands in ALSPAC have been measured previously in vivo, with high correlation between these measures and photocopies (r=0.97).^50^

### Confounding variables

We adjusted for the potential confounding effect of a number of sociodemographic measures. Each parent's self-reported occupation was coded as one of professional, managerial and technical, skilled non-manual, or manual,^51^ and the highest reported class from each child's parent(s) was recorded. Highest self-reported maternal education level was recorded as certificate of secondary education, vocational, ‘O’ level, ‘A’ level or degree. Occupational class and maternal education were used as a proxy for socioeconomic status (SES), which is known to predict loss to follow-up in ALSPAC.^38^ ASDs are socially patterned,^52^ although recent research suggests this may be in a direction opposite to that previously reported.^53^ We also adjusted for the age at which the participants attended the 11-year clinic (where the 2D:4D exposure was measured) to account for compliance to clinic invite, which may be related to the outcomes considered.

### Statistical analysis

**Figure 1:**
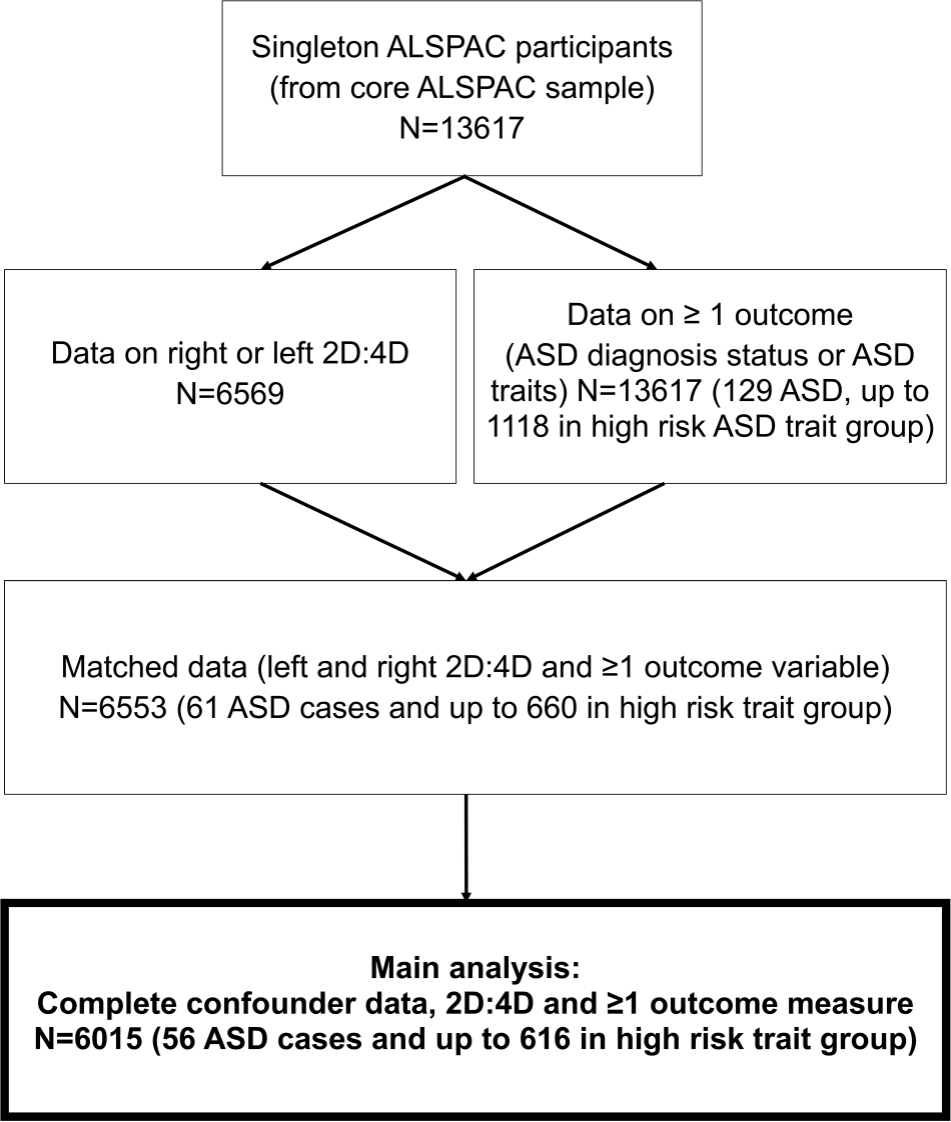
Description of case selection (ASD, autism spectrum disorders; ALSPAC, Avon Longitudinal Study of Parents and Children; 2D:4D, second-to-fourth digit ratio).

For the main analysis, we included children from singleton pregnancies with left and right 2D:4D measured, who had data for at least one of five outcome measures (recorded presence/absence of ASDs, or data on at least one of the four dichotomised ASD trait measures) and additionally, data on potential confounders, including parental occupational class, maternal education level and age at 2D:4D measurement (n=6015, including 56 ASD cases, and up to 616 in a high-risk dichotomised group; see figure 1 and table 1).

**Table 1:** Characteristics of the study sample compared with those ALSPAC participants not included in the study*

	Study sample (n=6015) (SDs given for means)	Other ALSPAC participants (n≤7602) (SDs given for means)	p Value†
Male (%)	49	53	≤0.001
Autism spectrum disorder (maternal and formal report) (%)	0.93	0.96	0.861
Mean 2D:4D (those with left and right 2D:4D, excluding two outliers)	0.964 (0.029)	0.963 (0.030)	0.421
Age at 2D:4D measurement, months	140.9 (2.8)	141.3 (3.2)	≤0.001
Manual occupational background (%)	14.2	25.3	≤0.001
Mother educated to degree level (%)	16.3	9.5	≤0.001

*Excluding multiple births, those not alive at 1 year, and those not in the core ALSPAC sample.

†From χ^2^ test for categorical variables, and unpaired t test (two-tailed) for continuous variables.

2D:4D, second-to-fourth digit ratio; ALSPAC, Avon Longitudinal Study of Parents and Children; n, number of participants.

**Figure 2:**
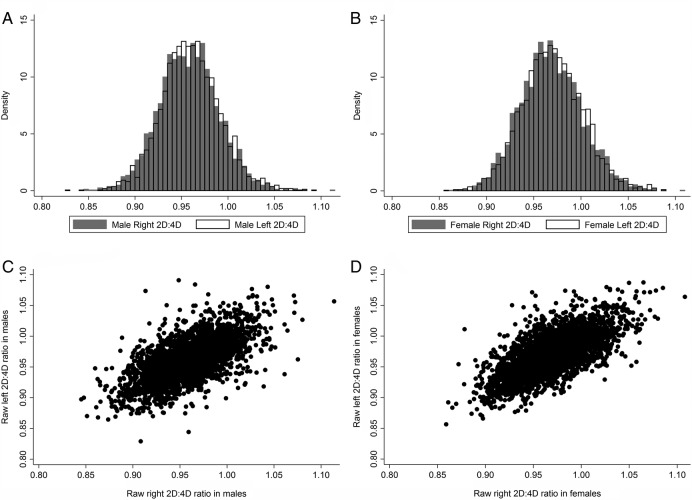
Histograms and scatterplots of left and right 2D:4D (second-to-fourth digit ratio), separately for males and females. (A) Histogram showing distribution of right and left 2D:4D in males. Mean (SD) of right 2D:4D: 0.958 (0.032); left 2D:4D 0.960 (0.032). (B) Histogram showing distribution of right and left 2D:4D in females. Mean (SD) of right 2D:4D 0.969 (0.033); left 0.970 (0.032). (C) Scatterplot of right and left 2D:4D in males; correlation between right and left hands: 0.64. (D) Scatterplot of right and left 2D:4D in females; correlation between right and left hands: 0.69.

Since the correlation between right and left hand 2D:4D was high (males 0.64, females 0.69), we used the mean of left and right hand 2D:4D in our main analysis (see figure 2). Two outliers were removed due to their extreme 2D:4D values (<0.75 and >1.25). Sex differences in this composite measure were assessed using a two-tailed t test. Given the male preponderance in ASDs, and since 2D:4D is sexually dimorphic, all analyses which followed were stratified by sex, in order to assess whether the relationship of 2D:4D and ASD varied between sexes. To enable a clearer comparison of the male and female results, the 2D:4D measure was further standardised within each sex, so that a one unit change in 2D:4D represents a change by 1 SD. Raw scores (ie, non-standardised scores) are presented for descriptive tables.

A univariable logistic regression model was estimated for each ASD-related outcome in turn, using the standardised mean 2D:4D variable as a continuous exposure (see table 3). Effects were subsequently adjusted for the potential confounders listed above.

A supplementary analysis adjusted for IQ (see online supplementary table S3). As sensitivity analyses, models were repeated using left and then right hand in turn (see online supplementary tables S4A and S4B). All analyses were carried out in Stata (StataCorp), V.13.

## Results

The study sample and those ALSPAC participants not in this particular study were similar in terms of mean 2D:4D, age at 2D:4D measurement and ASD prevalence, but fewer participants in our study were male (see table 1). Among our study sample, there was an excess of ASD cases identified from maternal report of diagnosis only, in comparison to cases identified by record linkage, despite the equivalent prevalence of ASDs between our study sample and the remaining ALSPAC participants (see table 1). Participants in the study sample were more likely to have parents working in non-manual occupations, were more likely to have a marginally higher IQ, and their mothers were more likely to be educated to degree level, which is consistent with the social patterning of attrition previously observed within ALSPAC (see table 1).^39^ Within the full sample (n=6015), there was strong evidence of sexual dimorphism for 2D:4D, with a mean (SE) of 0.959 (0.001) in males and 0.969 (0.001) in females (t=13.4, df=6013, two-tailed t test p≤0.001). This sexual dimorphism was still present in the subsample of ASD cases (n=56, males 2D:4D=0.955 (0.003); females=0.979 (0.006), t=3.5, df=54, p≤0.001). Mean 2D:4D values stratified by ASD diagnosis and the other outcomes are shown in table 2 (see online supplementary table S2 for results shown separately for right and left hand, and for results stratified by record linkage vs maternal report of ASD diagnosis).

**Table 2:** Mean (SE) of left and right second-to-fourth digit ratio, and numbers (n) in each subgroup (autism spectrum disorder (ASD)/no ASD; high/low ASD risk), by sex

			Males		Females	
Outcome	Age	Risk group	Mean (SE)	N	Mean (SE)	N
ASD diagnosis	Diagnosed by 11 years	No ASD diagnosis	0.959 (0.001)	2920	0.969 (0.001)	3039
		ASD diagnosis	0.955 (0.003)	42	0.979 (0.006)	14
Children's Communication Checklist (Coherence subscale)	115 months	Lower risk	0.959 (0.001)	2285	0.970 (0.001)	2495
		Higher risk	0.962 (0.002)	302	0.969 (0.002)	167
Social and Communication Developmental Checklist	91 months	Lower risk	0.960 (0.001)	2260	0.970 (0.001)	2444
		Higher risk	0.959 (0.002)	299	0.970 (0.002)	172
Repetitive behaviour	69 months	Lower risk	0.960 (0.001)	2378	0.970 (0.001)	2509
		Higher risk	0.958 (0.002)	199	0.969 (0.003)	130
Emotionality, Activity and Sociability Temperament Scale (Sociability subscale)	38 months	Lower risk	0.959 (0.001)	2378	0.970 (0.001)	2540
		Higher risk	0.959 (0.002)	352	0.968 (0.002)	264

Table 3 shows unadjusted and adjusted estimates for the increase in odds of each outcome per 1 SD increase in mean 2D:4D ratio. There was no evidence of an association between mean 2D:4D and any of the five outcomes in either sex.

**Table 3:** Logistic regression of ASD versus no ASD, or high-risk dichotomised trait versus low risk, against mean 2D:4D (left/right hands combined), stratified by sex

Sex	Males				Females			
	Unadjusted	Adjusted*			Unadjusted	Adjusted*		
Model	OR (95% CI) p value	OR (95% CI) p value	Total N	Risk N†	OR (95% CI) p value	OR (95% CI) p value	Total N	Risk N†
ASD diagnosis	0.87 (0.64 to 1.19) p=0.393	0.88 (0.65 to 1.21) p=0.435	2962	42	1.39 (0.83 to 2.34) p=0.206	1.36 (0.81 to 2.28) p=0.245‡	3053	14
Children's Communication Checklist (Coherence subscale)	1.10 (0.97 to 1.24) p=0.124	1.10 (0.98 to 1.24) p=0.118	2587	302	0.98 (0.84 to 1.15) p=0.790	0.98 (0.84 to 1.15) p=0.809	2662	167
Social and Communication Developmental Checklist	0.96 (0.85 to 1.09) p=0.553	0.96 (0.85 to 1.09) p=0.550	2559	299	1.03 (0.88 to 1.20) p=0.698	1.04 (0.89 to 1.22) p=0.601	2616	172
Repetitive behaviour	0.95 (0.82 to 1.10) p=0.466	0.95 (0.82 to 1.09)	2577	199	0.98 (0.82 to 1.17)	0.98 (0.82 to 1.17)	2639	130
		p=0.458			p=0.796	p=0.840		
Emotionality, Activity and Sociability (Sociability subscale)	0.98 (0.87 to 1.09) p=0.670	0.97 (0.87 to 1.08) p=0.583	2730	352	0.94 (0.83 to 1.07) p=0.325	0.94 (0.83 to 1.07) p=0.357	2804	264

Unadjusted and adjusted models are presented. NB ORs are for one unit change in Z score (ie, 1 SD).

*Adjusted for highest parental occupational class, maternal education and age at 2D:4D measurement.

†Risk N includes those with ASD diagnosis or in the high risk group for the ASD trait.

‡To avoid perfect prediction, the CSE and vocational classes of maternal education were collapsed for this analysis.

2D:4D, second-to-fourth digit ratio; ASD, autism spectrum disorder; CSE, certificate of secondary education; N, numbers of participants.

We included IQ as measured by a modified version of the Wechsler Intelligence Scale for Children, as an additional covariate in a supplementary analysis. We considered this separately from our main analysis to prevent a substantial loss to our sample size, since the IQ measure was available on only 44/56 of included participants with ASDs. Another supplementary analysis examined the effect of 2D:4D by left and right hand.

Supplementary tables show (1) little impact of further adjustment for IQ (see online supplementary table S3), and (2) a broadly consistent pattern of findings when examining the effect of 2D:4D ratio for each hand in turn (see online supplementary tables S4A and S4B). One subanalysis suggested a weak association between reduced coherence (as measured by the Children's Communication Checklist) with left 2D:4D in males (adjusted OR 1.15 (95% CI 1.02 to 1.29), p=0.023, see online supplementary table S4A). There was also a trend towards increased right 2D:4D being associated with ASD diagnosis in females (adjusted OR 1.60 (95% CI 0.97 to 2.65), p=0.068, see online supplementary table S4B). However, these results are in the opposite direction to that predicted by the prevailing theory of the EMB. One subanalysis found a trend towards masculinised 2D:4D being associated with the repetitive behaviours measure OR 0.87 (95% CI 0.75 to 1.01), p=0.061. Yet, given the number of comparisons and differing directions of association, we consider that these results are consistent with chance.

## Discussion

In this large UK-based birth cohort, we did not find evidence to support the hypothesis that lower 2D:4D is associated with an increased risk of ASDs. We also examined the relationship of digit ratio with dichotomised component trait measures of ASDs, including social cognition, coherence, repetitive behaviours and sociability. There were several suggestions of very weak relationships between 2D:4D and ASD traits, when the hands were studied separately. These associations were mostly directionally discordant with the extreme male theory of autism, and given the number of comparisons made, and the lack of consistent association between ASD diagnosis and 2D:4D in this study, we consider that they were due to chance.

Recent reviews have concluded that there is support for an association between masculinised 2D:4D and ASD.^37^ ^38^ The central estimate from the most recent quantitative review was a Cohen's d of −0.43, representing a moderately lower digit ratio among individuals (mostly males were studied) with autism.^38^ Using a t test (comparing 2D:4D between males with and without ASD—the most common comparison in the literature), we calculated that we would have had 79% power to detect this central estimate. However, we chose logistic regression methods for our primary analysis rather than a t test, since we were interested in adjusting for the effect of potential confounders. For consistency with previous literature, we repeated our analyses (mean 2D:4D in males with and without ASD) using a t test, and found a Cohen's d of −0.13 (95% CI −0.44 to 0.17). It should be noted that the d of −0.43, reported in the literature^38^ is within the CI of this estimate, albeit at the extreme lower bound.

Among the studies examining the 2D:4D–ASD diagnosis relationship (ie, not ASD trait measures), most studies recruited cases from clinical settings,^17^ ^21–23^ which may potentially limit the external validity of results, if cases recruited in a clinical setting are qualitatively different to the larger population from which they are sampled. Others recruited from mixed clinical/non-clinical populations,^16^ ^25^ schools^18^ ^20^ and a charity.^15^ ASD case numbers in these studies varied from 23^20^ to 216.^25^ Of two studies with the largest number of cases to date, one was a population-based study, and these authors found an association between masculinised 2D:4D and ASD.^15^ Another was a clinically recruited sample, which found no relationship between 2D:4D and ASD.^25^ Overall, results of published studies examining the 2D:4D–ASD relationship have therefore been mixed, although the majority of them have reported an association concordant with the EMB theory.^15^ ^17^ ^19–22^ Others have found no association,^18^ ^23^ ^25^ or the inverse association to the prevailing hypothesis.^16^ It is also notable that many studies have examined case populations that are entirely or almost entirely male.^17–20^ ^23^

Although most case–control studies selected age-matched and sex-matched controls, the possibility remains that the composition of the ALSPAC population may differ from these reference populations. The strengths of this study are that it is population-based, which is a design that is generally less susceptible to selection bias. We analysed the association between 2D:4D and ASD risk separately by sex, which may be important, since 2D:4D is a sexually dimorphic trait. The population studied had a comparatively high proportion of female ASD cases; however, despite this, the absolute number of female ASD cases in our study was also small (n=14). Although 2D:4D is sexually dimorphic, it has been found that this dimorphism may not be apparent in individuals with ASD.^15^ In our study, 2D:4D was sexually dimorphic in individuals with or without ASD. Further strengths of this study are that we were able to study various component measures of ASD in a large population.^37^ ^54^ Although one large study has found an association between 2D:4D and ASD traits,^54^ reviews of previous literature have asserted that there is little overall evidence for the relationship between 2D:4D and ASD traits in healthy participants.^37^ ^38^ Our study also found no consistent association between 2D:4D and ASD trait measures.

The ALSPAC cohort provides a rich resource of data, from which we selected variables that may have confounded an association between 2D:4D and ASD. Although our sample was population-based, the possibility of selection bias in relation to baseline recruitment or attrition in the sample over time cannot be entirely excluded. We tried to address this possible attrition bias by controlling for the characteristics known to be predictive of attrition in ALSPAC, such as SES. However, it is also possible that those children severely affected by ASD may have been less likely to attend clinics where 2D:4D was measured—this could therefore result in our sample being weighted towards Asperger syndrome and other high-functioning ASD cases. This may be relevant given that previous studies have observed a less profound difference in 2D:4D in children with Asperger syndrome compared with ASD.^15^ Finally, although the method used to measure 2D:4D has been shown to be reliable,^50^ ^55^ and used by some of the previous studies on this topic,^15^ ^17^ the possibility of error in measurement of 2D:4D cannot be excluded. Yet, it is unlikely that this would be differential for people with and without a diagnosis of ASD.

One recent study reported an association between fetal steroidogenic activity (including testosterone) and ASD, providing some direct evidence in support of the biological basis of the EMB theory of autism.^14^ Yet, as fetal testosterone cannot be readily measured, papers assessing the relationship between fetal testosterone and ASDs, including our study, require the existence of a proxy, for example, 2D:4D. One study cites direct evidence of 2D:4D being related to fetal testosterone-to-oestradiol ratio in humans.^56^ Some of the strongest indirect evidence for the fetal testosterone–2D:4D relationship comes from studies associating 2D:4D with increased (congenital adrenal hyperplasia)^28^ ^34^ ^36^ and decreased (complete androgen insensitivity syndrome)^35^ endocrine models of fetal testosterone exposure. Animal studies have provided mixed results, with some in favour of the fetal testosterone–2D:4D relationship,^57^ ^58^ and others supporting the inverse association,^59^ or no association.^60^ Studies of repeats in the androgen receptor gene (correlated to testosterone) and 2D:4D have similarly provided mixed results.^50^ ^61–63^ A genome-wide scan (including ALSPAC participants) found the minor allele of one single nucleotide polymorphism in LIN28B (linked to delayed age at menarche)^64^ to be associated with increased 2D:4D.^50^ This association was in the opposite direction to that predicted from previous work, in which lower 2D:4D was associated with delayed age at menarche, putatively via higher fetal testosterone exposure.^65^ The authors therefore suggested that the relationship between fetal testosterone and 2D:4D may be more complex than first proposed, which is in agreement with previous work.^56^ However, regardless as to whether 2D:4D ratio is mainly a marker of fetal testosterone, 2D:4D is clearly sexually dimorphic and related to hormonal traits within sexes,^65^ and thus may still be useful in the study of other sex-related traits.

To conclude, in this large population-based study, we found no consistent evidence supporting an association between lower 2D:4D ratio and increased risk of ASDs, or ASD component traits. These findings are not consistent with the EMB theory of autism.
